# Intra-Population Variation of Secondary Metabolites in *Cistus ladanifer* L.

**DOI:** 10.3390/molecules21070945

**Published:** 2016-07-21

**Authors:** Cristina Valares Masa, Juan Carlos Alías Gallego, Natividad Chaves Lobón, Teresa Sosa Díaz

**Affiliations:** Department of Plant Biology, Ecology and Earth Sciences, Faculty of Science, University of Extremadura, 06006 Badajoz, Spain; cvalmas@unex.es (C.V.M.); jalias@unex.es (J.C.A.G.); natchalo@unex.es (N.C.L.)

**Keywords:** flavonoids, diterpenes, intrapopulation variation, spatial heterogeneity

## Abstract

In previous studies, secondary metabolites in the leaf exudate of *Cistus ladanifer*, specifically aglycone flavonoids and diterpenes, were demonstrated to play an ecophysiological role. They protect against ultraviolet radiation, have antiherbivore activity, and are allelopathic agents. Their synthesis in the plant was also found to vary quantitatively and qualitatively in response to various environmental factors. In view of these findings, the present work was designed to clarify whether within a single population there are differences among individuals subject to the same environmental conditions. To this end, we analyzed the leaves of 100 individuals of *C. ladanifer*. The results showed the existence of intrapopulational variation, since, although all the individuals had the same composition of secondary chemistry, the amounts were different. The individuals of a given population of *C. ladanifer* differ from each other even when growing under similar conditions. According to the ammount of flavonoids and diterpenes observed in each individual, it was possible to distinguish four different groups of individuals. Most individuals, evenly distributed within the population, had low concentrations of the studied compounds, whilst other individuals synthesized greater amounts and were randomly distributed among the former. Given the functions of flavonoids and diterpenes in this species, the quantified intra-population variation may involve greater plasticity for the species in the face of environmental changes.

## 1. Introduction

Many plants have defenses against a wide range of stress factors and their variations in defense may depend on the concentration of secondary metabolites. Like most traits of organisms, plant secondary metabolites are variable at all scales including among species, populations, individuals, and different plant parts [1,2]. Strong intraspecific variation in defense chemical concentration or composition occurs in many plant species [3,4]. This fact suggests that the chemical complexity of a plant species within a population could provide protection against different stress factors. Within a given population, environmental conditions are very homogeneous [5]. This way, intra-individual and intra-population variation in the accumulation of secondary metabolites may represent a component of phenotypic variability with an important adaptive value [6]. In fact, it has been demonstrated that anthocyanins and other flavonoids usually accumulate transiently in vegetative tissues, as a plastic response to biotic or abiotic stressors [7,8].

*Cistus ladanifer* L. (“rockrose”) is a shrub that secretes a large amount of exudate from its leaves and photosynthetic stems [9]. This exudate is secreted by trichomes [10] and it is characterised by two families of secondary metabolites: flavonoids (apigenin, kaempferol 3-methyl ether, apigenin 4′-methyl ether, apigenin 7-methyl ether and kaempferol 3,7-di-*O-*methyl ether) [11], which constitute between 6% and 26% of the dry weight of the exudate [12], and diterpenes (6β-acetoxy-7-oxo-8-labden-15-oic acid, 7-oxo-8-labden-15-oic acid, oxocativic acid), which constitute between 1% and 2% of the dry weight of the exudate [13,14].

In previous studies, we have shown that the flavonoids and diterpenes in the exudate of *Cistus ladanifer* L. leaves play an important ecophysiological role. They protect against ultraviolet radiation, have antiherbivory activity, and are allelopathic agents [13,15,16,17]. The synthesis of these compounds in the plant varies quantitatively and qualitatively in response to such exogenous factors as photoperiod, ultraviolet light, temperature, and water stress [18], and the interpopulation variation in the quantity of these compounds has been shown to be dependent on climatic conditions [12]. Recent research works [19] show that there are significant differences between young leaves, mature leaves and stems, and between individuals of different ages. In addition, the variation in the composition of secondary metabolites between different parts of the plant, the season and the variations in age may determine the interactions between *Cistus ladanifer* and its environment [20].

The information about the metabolic profile of plants exudates at the population level is scarce. Jandová et al. [21] found a strong evidence that intraspecific variation needs to be considered in the research on allelopathy, and suggest that metabolic profiling provides an efficient tool for studying chemically mediated plant–plant interactions whenever unknown metabolites are involved.

Therefore, given the function of flavonoids and diterpenes in this species, the objective of the present work was to study the possible intra-population variation of the defense metabolites in the composition of flavonoids and diterpenes in *C. ladanifer* leaves.

## 2. Results

### 2.1. Secretion of Secondary Metabolites

**Figure 1:**
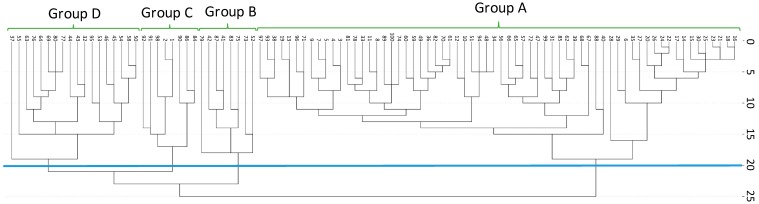
UPGMA dendrogram of the chemical similarity among the plant individuals within the study population (*n* = 100).

**Figure 2:**
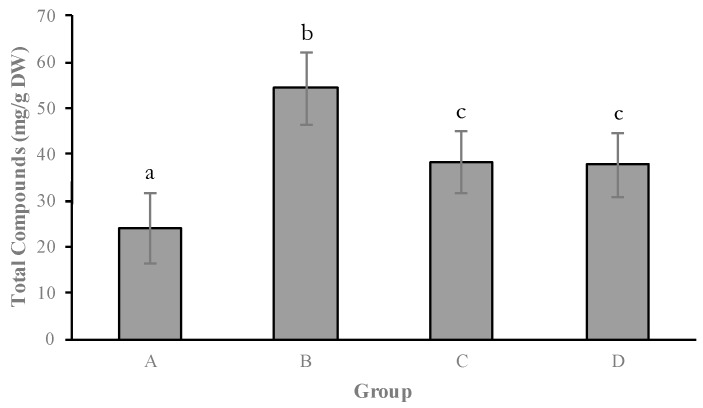
The groups denote to order of UPGMA clustering for 100 individual plants. Amount of Total Compounds (mg/g DW) in different groups. a, b, c: differences in small letters indicate significant differences between groups (*p* < 0.05). Bars indicate the standard error.

**Figure 3:**
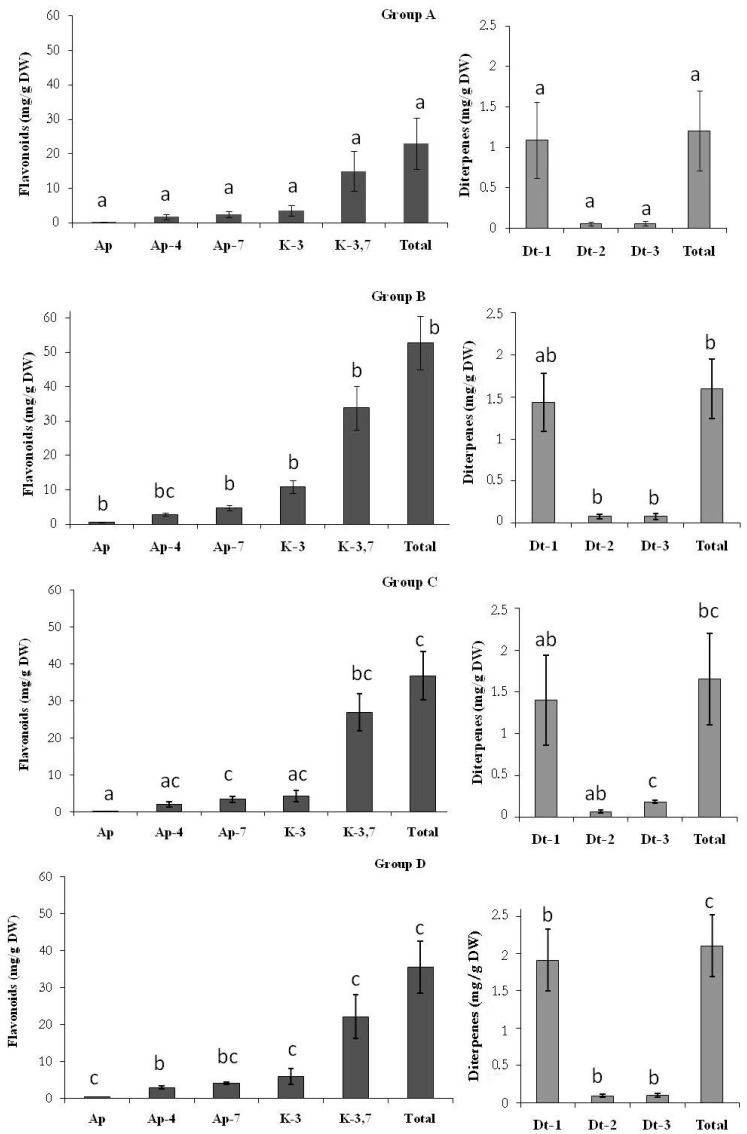
The groups denote to order of UPGMA clustering for 100 individual plants Amount of flavonoids and diterpenes (mg/g DW) in different groups. Ap: apigenin; Ap-4: apigenin 4′-methyl ether; Ap-7: apigenin 7-methyl ether K-3: kaempferol 3-methyl ether; K-3,7: kaempferol 3,7-di-*O*-methyl ether; Dt-1: 6β-acetoxy-7-oxo-8-labden-15-oic acid; Dt-2: 7-oxo-8-labden-15-oic acid; Dt-3: oxocativic acid. a, b, c: differences in small letters indicate significant differences between groups (*p* < 0.05). Bars indicate the standard error.

The main phenolic compounds present in the individuals of *C. ladanifer* studied were flavonoids (apigenin, kaempferol 3-methyl ether, apigenin 4′-methyl ether, apigenin 7-methyl ether and kaempferol 3,7-di-*O-*methyl ether) and diterpenes (6β-acetoxy-7-oxo-8-labden-15-oic acid, 7-oxo-8-labden-15-oic acid and oxocativic acid) (Table S1 in Supplementary Materials). Kaempferol 3,7-di-*O-*methyl ether was the most abundant chemical species of the flavonoids group, and oxocativic acid regarding the diterpenes group. The results showed that the variation in secondary metabolite secretion among the analysed individuals was quantitative. Four distinct chemotypes (groups A–D) were found among the 100 studied plants by UPGMA clustering (Figure 1). Generally, this cluster highlights that the values of secondary metabolite concentration (Figure 2 and Figure 3) ranged between 52.74 mg/g PS (group B) and 23.00 mg/g PS (group A) in flavonoids and 2.10 mg/g PS (group D) and 1.21 mg/g PS (group A) in diterpenes, being these variations significant. According to this cluster (Figure 1), 66% of the individuals constituted group A. The individuals of group A (Figure 3) synthesize the lowest concentrations of all flavonoids (23.00 mg/g PS) and diterpenes (1.21 mg/g PS). Group B was formed by 8% of the individuals, whose chemical profile corresponded to the highest concentrations of flavonoids (54.72 mg/g PS) and moderate concentrations of diterpenes (1.60 mg/g PS). A third group (C) was made also by 8% of the individuals. These showed low amounts of all flavonoids (except kaempferol 3,7-di-*O-*methyl ether, which presented high levels) and moderate amounts of diterpenes (oxocativic acid, showing the greatest amount of all groups (0.2 mg/g PS). Finally, group D, formed by 18% of the individuals, showed moderate concentrations of flavonoids and the highest concentrations of diterpenes, with the amount of 6β-acetoxy-7-oxo-8-labden-15-oic acid being greater than that of the rest of the groups (1.93 mg/g PS). Generally, apigenins presented significant differences between groups A and B, kaempherols among groups A, B and D, and diterpenes showed significant differences in at least two of the groups (A and D).

### 2.2. Spatial Distribution of the Individuals of the Studied Population

**Figure 4:**
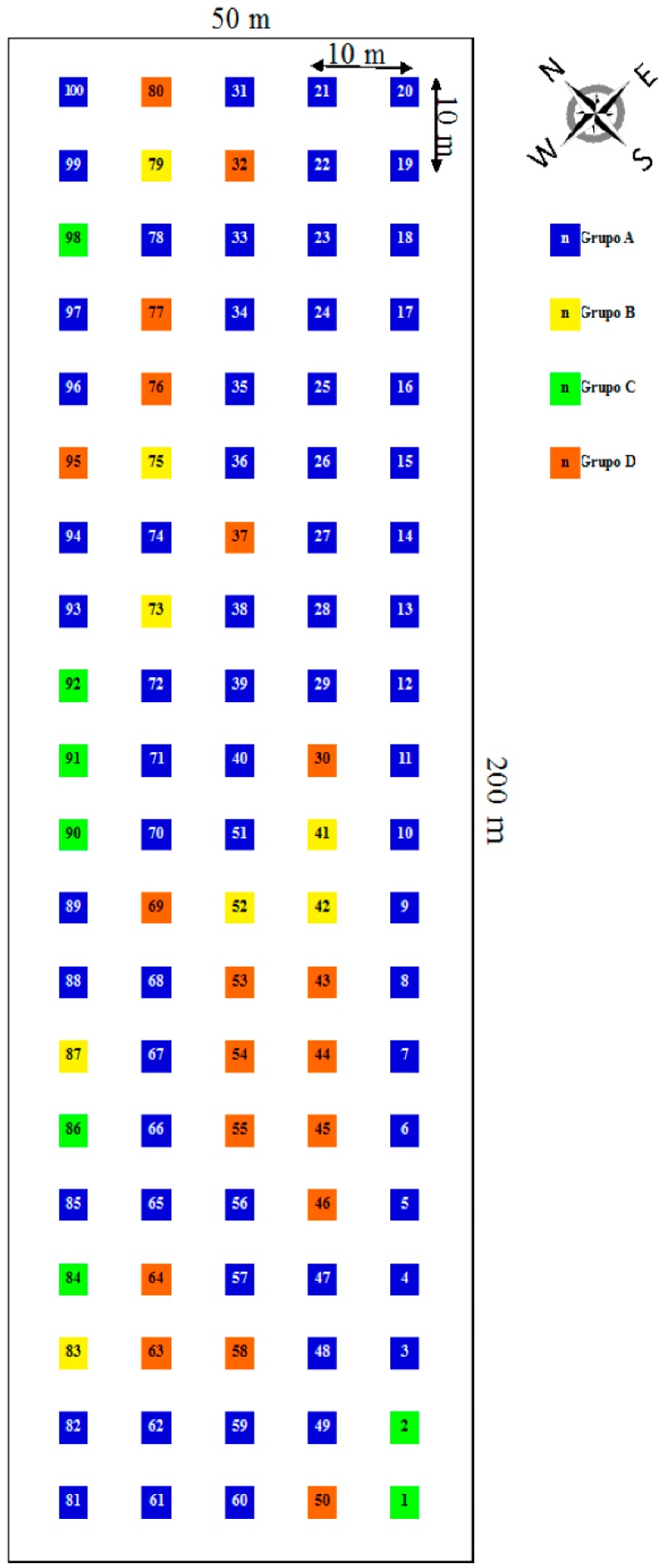
Spatial distribution of different individuals of *Cistus ladanifer* sampled, n: individual (*n* = 100).

From the distribution observed (Figure 4) it is important to highlight that most of the individuals that constitute the studied population (66%) showed low amounts of all the studied compounds, being evenly distributed over the area occupied by the population. On the other hand, the individuals that showed greater levels in most of the compounds (group B) and those with moderate amounts (C and D) were distributed among those with lower amounts (group A). B and C individuals were randomly distributed and those of group D formed groups. This remark indicates that the spatial distribution of the different individuals causes the high diversity of the population.

## 3. Discussion

Flavonoids and diterpenes exert a wide variety of ecological and physiological functions [16,17,22,23,24,25,26,27,28,29,30,31,32]. The properties of these compounds depend on their concentration, and the concentration depends on the synthesis of these compounds in the plant. In *C. ladanifer*, the synthesis of flavonoids and diterpenes depends on the effect of environmental factors, such as ultraviolet light, photoperiod and water stress [12,13,14,15,19,33]. With this new study, the synthesis of these compounds was quantified in the individuals of a population under the same external factors. The intra-population variation found shows that the synthesis of these compounds is conditioned not only by environmental factors but it may also vary from one individual to another. Our results showed a high degree of spatial variation in the leaf concentration of phenols and terpenes within the *C. ladanifer* population. There is a clear quantitative variation among the individuals that constitute the studied population.

Furthermore, the results demonstrate that the individuals of a population can be grouped based on their amount of flavonoids and diterpenes. Even though the Cluster Analysis performed is an artificial method, it is a useful tool for the elaboration of at obtaining a global view of the variation of these compounds within a population. Few papers have described this variation at a scale range of meters to hundreds of meters. The results showed that there is was a great spatial variation in the secretion of secondary metabolites in the leaves of *C. ladanifer*. The individuals of different groups were mixed and randomly distributed. This variability and spatial arrangement may be a strategy to save energy while preserving the response capacity and protection against environmental factors.

Vourc’h et al. [34] explained the variation of plant defenses inside a population as a consequence of the interaction of the environmental variability of plant resources, genotypic differences among individuals, and herbivore or pathogen effects. These three causes of variability may be operating in our study population. Provided that the interaction among species takes place in space, the spatial pattern of the compounds of secondary metabolism may influence any biological process, affecting their concentration. The spatial structure of the secondary metabolites of leaves may be relevant, for example, for plant-herbivore interaction, since it may create spatial attack patterns of herbivores to plants [35,36] and slow down the adaptation of the herbivore to the defense compounds of the plant [35,37]. According to what was suggested by Karban et al. [35], the responses induced toward herbivory increase the temporal and spatial variability of the secondary metabolites of plants.

Diversity can be inherently beneficial for plant individuals, families, populations and communities. Intraspecific variation in plant secondary metabolites was a formative example in developing the concepts of community and ecosystem genetics [38].

Our results indicate that in a natural “rockrose” formation the variation in defense chemistry is high, which gives good possibilities for populations to adapt to variable environmental effects. The presence of intra-specific variation would be a clear advantage over another species with more similarity among the individuals of one population. With greater variation, the species is more likely to survive changes due to the high probability to produce an individual with the adequate amount of compounds to respond to those changes [39,40]. Trade-offs related to the production of allelochemicals in *C. ladanifer* need to be estimated in order to explain why the variability is sustained in a system.

## 4. Materials and Methods

### 4.1. Description of C. ladanifer

*C. ladanifer* L. is a typically Mediterranean species [41,42,43] that is widely distributed over the western Iberian Peninsula. It is a perennial shrub with a height range of 1–4.5 m that occupies highly disturbed areas associated with early successional stages. It shows great efficacy in adapting to unfertile soils [41,42], and it forms shrubby communities that are often nearly monospecific and highly persistent [44].

### 4.2. Study Area and Sample Collection

The site chosen for the collection of material was a rockrose formation (a stand of *C. ladanifer* L.) located in the municipality of Alburquerque (South-west Spain) (39°09′38.30″ N, 6°58′43.02″ W). The area is situated at 289 m a.s.l. and had Mediterranean climate. Average annual rainfall: 500 mm^3^; December and January are the months with highest rainfall and July and August had the lowest rains [45]. The vegetation of the area is shrubland, dominated by *C. ladanifer*. Voucher specimens were deposited at the Herbarium of University of Extremadura: UNEX 36259.

**Figure 5:**
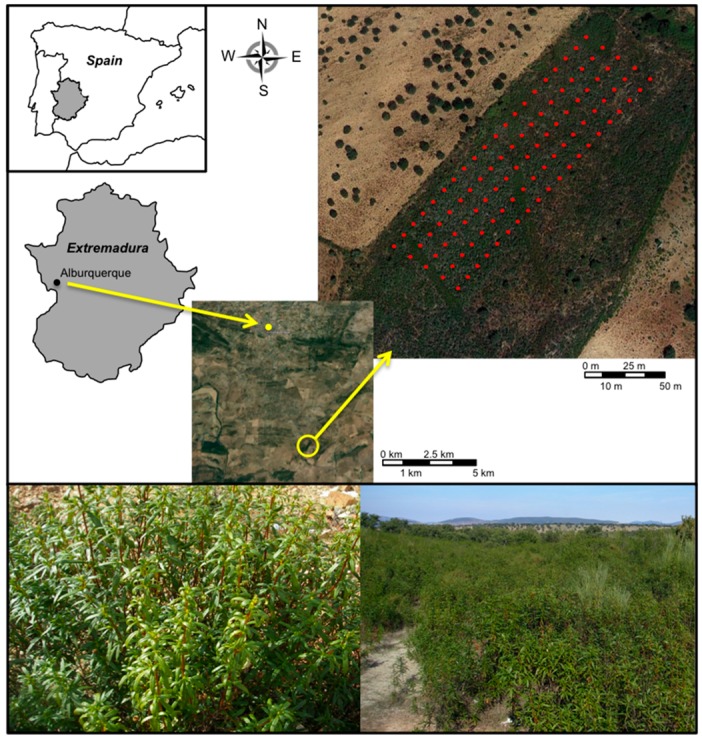
Location of the study area. Each red dot represents an individual. A view of an individual and a shrubland dominated by rockrose *C. ladanifer*.

The sampling area was 200 m × 50 m (10,000 m^2^) (Figure 5). This area showed homogenous climatic and edaphological characteristics, with nutrient-poor soils. In this area, 100 individuals of *C.* *ladanifer* were selected (individuals of 20 mm in trunk base diameter: approximately 12 years of age according to the equation y = 1.5496x + 1.5342; R^2^ = 0.9221 [46]. The individuals were separated from each other by a distance of 10 m.

In studies performed by Valares [46] and Valares et al. [19], it was shown that the amount of compounds derived from the secondary metabolism present in one individual of *C. ladanifer* depends upon the season and the type and development state of the plant organ. Young leaves (sprouts at the apical part), collected during the summer, autumn and winter, are the organs in which the lowest variation is quantified. Thereby, this was the type of leaf selected for the quantification of intrapopulation variation.

At the beginning of autumn (4 October 2010) three sprouts of young leaves (4–6 leaves per sprout) were collected from each individual (all samples were collected on the same day). The samples were individually packed in aluminium foil in situ, numbered and then stored in bags in a refrigerator. Immediately after collection, the plant material was transported to the laboratory and the exudate of each sample (*n* = 300) was extracted at the sampling day.

### 4.3. Extraction and Assay of Secondary Metabolites

**Figure 6:**
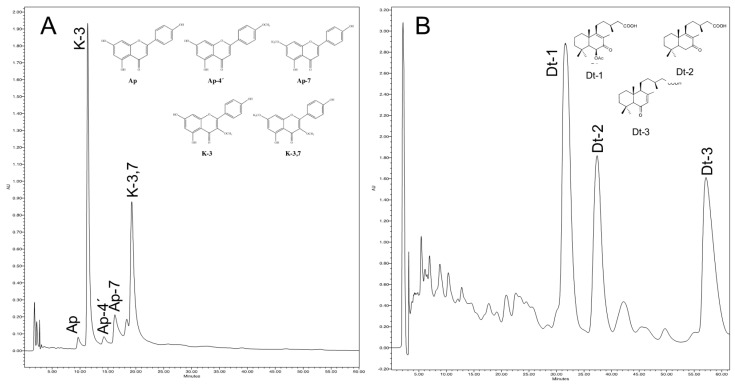
Chemical structures and HPLC chromatograms at 350 nm of flavonoids (**A**): Ap: apigenin; Ap-4′: apigenin 4′-methyl ether; Ap-7: apigenin 7-methyl ether K-3: kaempferol 3-methyl ether; K-3,7: kaempferol 3,7-di-*O*-methyl ether; and at 250 nm of diterpenes (**B**): Dt-1: 6β-acetoxy-7-oxo-8-labden-15-oic acid; Dt-2: 7-oxo-8-labden-15-oic acid; Dt-3: oxocativic acid extracted from leaves of *C. ladanifer*.

Approximately 1 g of young leaves were weighed out. The exudate was extracted with chloroform at a ratio of 1:10 *wt*:*v* to ensure complete extraction of flavonoids and diterpenes [15]. The chloroform was evaporated off under a fume hood at a maximum temperature of 30 °C and the pellet was resuspended in 4 mL of methanol. The resulting solution was maintained frozen at −20 °C for 12 h to precipitate out the waxes which were then removed by centrifugation and the supernatant was stored at 4 °C for its subsequent analysis [47]. The secondary metabolites were analysed by HPLC (Pumps: 515 HPLC Pump; 717-plus Autosampler Injector; 996 Photodiode Array Detector, Waters, Milford, MA, USA). 80 µL of each sample were injected into a Spherisorb C-18, 5 µm, 4.6 × 250 mm reverse phase analytical column. The mobile phase used was water/methanol/tetrahydrofuran in the proportion 56/16/28 at a 0.75 mL/min flow rate. The chromatograms were recorded at absorbance maximum wavelength of 350 nm for flavonoids and 250 nm for diterpenes (Figure 6). These conditions yield a chromatogram with optimal resolution for the identification of the five flavonoids [9,15,48] and three diterpenes [13] present in the exudate of *C. ladanifer*. The compounds were identified on the basis of their retention times and spectral characteristics [9,15,48]. In order to obtain the flavonoids and diterpenes and to determine the linear calibration equation, the following criteria and methodology were adopted: the flavonoids and diterpenes identified in *C. ladanifer* are not commercially available and were therefore extracted from the exudate. The exudate was extracted with chloroform. For the separation of the flavonoids and diterpenes, the extract was dissolved in hot methanol (10 mL/mg extract) [47], and the solution stored at −20 °C for 12 h, causing the precipitation of methanol-insoluble compounds, mainly hydrocarbons and waxes. The supernatant was directly chromatographed on Sephadex LH-20 (12.5 g) on a 25 cm long~1.5 cm diameter column, with methanol as the eluent. With this procedure, the flavonoids are separated from the diterpenoids and the other phenolics. Several extracts were obtained, and these were subsequently analysed by HPLC to determine the fraction containing the flavonoids and diterpenes. The fraction with flavonoids was separated by using a semipreparative Nucleosil C-18, 5 μm (250 Å~10 mm) column and a water–methanol–acetonitrile–tetrahydrofuran (56:16:6:22) solution at a flow rate of 1.75 mL/min. Flavonoids were detected with diode array at 350 nm wavelength. As the peak of each flavonoid was detected, the fraction was collected in a separate tube. To eliminate any possible contamination from other compounds eluting close to the flavonoid being purified, the fraction was again separated by HPLC with a methanol–water (80:20) solvent at a 2.5 mL/min flow rate.

The flavonoids purified from the extract were: apigenin, kaempferol 3-methyl ether, apigenin 4′-methyl ether, apigenin 7-methyl ether and kaempferol 3,7-di-*O-*methyl ether [11].

The fraction with diterpenes was separated by using a semipreparative Nucleosil C-18, 5 μm (250 Å~10 mm) column and a water-methanol-tetrahydrofuran (40:30:30) solution at a flow rate of 1.75 mL/min. They were detected with a diode array at 250 nm wavelength. As the peak of each diterpene was detected, the fraction was collected in a separate tube. To eliminate any possible contamination from other compounds eluting close to the diterpene being purified, the fraction was again separated by HPLC with a methanol–water (80:20) solvent at a 2.0 mL/min flow rate. The diterpenes purified from the extract were: 6β-acetoxy-7-oxo-8-labden-15-oic acid, 7-oxo-8-labden-15-oic acid and oxocativic acid [13]. The results obtained were expressed with reference to the leaves dry weight.

### 4.4. Statistical Analyses

Inter-Group Linkage was chosen as the Clustering Method, using the Average: Unweighted Pair Group Method using Arithmetic averages (UPGMA). The dissociative Hierarchical Method was selected as the Cluster Method and Euclidean Distance as the association measure. Eventually, the variable was standardised using Z-scores. Due to the normality of the data, an ANOVA test (SPSS-Win 15.0) was used to determine significant differences among different groups and the Bonferroni’s correlation to establish which groups were significantly different (significance *p* ≤ 0.05).

## Supplementary Materials

Supplementary materials can be accessed at: http://www.mdpi.com/1420-3049/21/7/945/s1.
