# Supplementary material for: Intra-Population Variation of Secondary Metabolites in Cistus ladanifer L
Source: Molecules. 2016 Jul 21;21(7):945. doi: 10.3390/molecules21070945 (PMC6274576; doi:10.3390/molecules21070945)
Supplement: Supplementary file 1 [file molecules-21-00945-s001.pdf]

Supplementary Materials: Intra-Population Variation of Secondary Metabolites in *Cistus ladanifer* L.

Cristina Valares Masa, Juan Carlos Alías Gallego, Natividad Chaves Lobón and Teresa Sosa Díaz

Table S1. Total flavonoids and diterpenes (mg/g DW) in exudate in a population of *Cistus ladanifer*, mean, SD, n=100. Ap: apigenin; Ap-4: apigenin 4′-methyl ether; Ap-7: apigenin 7-methyl ether; K-3: kaempferol 3-methyl ether; K-3,7: kaempferol 3,7-di-*O*-methyl ether; Dt-1: 6β-acetoxy-7-oxo-8-labden-15-oic acid; Dt-2: 7-oxo-8-labden-15-oic acid; Dt-3: oxocativic acid.

Individual	Ap	Ap-4	Ap-7	K-3	K-3.7	Dt-1	Dt-2	Dt-3	Total Compounds	Total Flavonoids	Total Diterpenes
Group A											
3	0.11 ± 0.01	1.09 ± 0.03	1.68 ± 0.09	1.94 ± 0.11	23.01 ± 1.63	0.93 ± 0.09	0.07 ± 0.01	0.11 ± 0.03	28.95	27.84	1.11
4	0.11 ± 0.01	0.86 ± 0.07	1.85 ± 0.20	2.00 ± 0.27	20.79 ± 1.58	1.32 ± 0.21	0.09 ± 0.01	0.09 ± 0.01	27.10	25.61	1.50
5	0.17 ± 0.05	1.45 ± 0.13	2.58 ± 0.07	2.27 ± 0.22	18.69 ± 1.03	1.25 ± 0.21	0.08 ± 0.01	0.08 ± 0.00	26.57	25.15	1.42
6	0.09 ± 0.03	0.91 ± 0.08	1.59 ± 0.06	0.64 ± 0.02	8.55 ± 2.67	1.07 ± 0.09	0.05 ± 0.01	0.03 ± 0.01	12.93	11.78	1.14
7	0.17 ± 0.03	1.35 ± 0.12	2.82 ± 0.17	2.19 ± 0.11	18.20 ± 2.35	1.38 ± 0.23	0.07 ± 0.02	0.06 ± 0.00	26.24	24.73	1.51
8	0.27 ± 0.08	1.25 ± 0.19	1.99 ± 0.07	4.64 ± 0.22	19.52 ± 4.81	1.11 ± 0.19	0.07 ± 0.03	0.05 ± 0.04	28.91	27.67	1.24
9	0.18 ± 0.03	1.12 ± 0.12	1.83 ± 0.04	3.00 ± 0.23	14.76 ± 3.13	1.25 ± 0.06	0.06 ± 0.01	0.05 ± 0.01	22.26	20.90	1.36
10	0.27 ± 0.05	1.03 ± 0.10	3.56 ± 0.13	4.18 ± 0.27	25.14 ± 2.85	1.32 ± 0.08	0.08 ± 0.01	0.09 ± 0.01	35.68	34.19	1.50
11	0.32 ± 0.09	1.83 ± 0.58	2.46 ± 0.15	3.55 ± 0.18	18.20 ± 2.88	1.10 ± 0.06	0.06 ± 0.01	0.05 ± 0.01	27.57	26.36	1.21
12	0.17 ± 0.04	0.98 ± 0.17	4.28 ± 0.67	4.49 ± 0.26	28.81 ± 3.38	1.35 ± 0.06	0.07 ± 0.01	0.09 ± 0.01	40.25	38.74	1.51
13	0.19 ± 0.06	0.89 ± 0.17	3.48 ± 0.17	2.98 ± 0.10	16.93 ± 3.68	0.72 ± 0.04	0.06 ± 0.01	0.08 ± 0.03	25.33	24.47	0.86
14	0.11 ± 0.02	0.82 ± 0.07	1.75 ± 0.08	2.87 ± 0.09	15.59 ± 2.87	0.65 ± 0.05	0.04 ± 0.01	0.05 ± 0.02	21.89	21.15	0.74
15	0.11 ± 0.01	1.41 ± 0.42	1.15 ± 0.17	3.41 ± 0.17	10.46 ± 2.16	0.53 ± 0.04	0.02 ± 0.00	0.04 ± 0.02	17.14	16.55	0.59
16	0.13 ± 0.03	0.73 ± 0.03	1.35 ± 0.06	3.92 ± 0.13	13.18 ± 3.13	0.64 ± 0.04	0.03 ± 0.00	0.03 ± 0.02	20.00	19.30	0.70
17	0.09 ± 0.02	0.52 ± 0.05	1.39 ± 0.17	3.18 ± 0.10	16.00 ± 3.36	0.51 ± 0.02	0.03 ± 0.01	0.05 ± 0.02	21.77	21.18	0.59
18	0.13 ± 0.02	0.86 ± 0.10	1.41 ± 0.20	3.71 ± 0.21	11.25 ± 1.20	0.61 ± 0.09	0.02 ± 0.00	0.04 ± 0.01	18.03	17.36	0.67
19	0.19 ± 0.03	0.81 ± 0.12	3.13 ± 0.16	3.31 ± 0.19	14.30 ± 3.37	0.60 ± 0.03	0.05 ± 0.01	0.06 ± 0.01	22.44	21.74	0.71
20	0.05 ± 0.01	0.51 ± 0.06	0.69 ± 0.03	1.13 ± 0.04	4.41 ± 0.41	0.33 ± 0.07	0.02 ± 0.00	0.01 ± 0.01	7.15	6.79	0.36
21	0.14 ± 0.05	0.96 ± 0.13	1.61 ± 0.19	2.70 ± 0.06	9.65 ± 0.85	0.49 ± 0.07	0.03 ± 0.01	0.03 ± 0.02	15.61	15.06	0.55
22	0.08 ± 0.00	0.83 ± 0.05	0.92 ± 0.04	1.94 ± 0.03	6.34 ± 0.92	0.39 ± 0.05	0.02 ± 0.00	0.02 ± 0.00	10.55	10.11	0.44
23	0.11 ± 0.03	0.95 ± 0.11	1.68 ± 0.15	2.54 ± 0.07	8.28 ± 0.84	0.57 ± 0.08	0.03 ± 0.02	0.04 ± 0.01	14.19	13.55	0.64
24	0.07 ± 0.01	0.63 ± 0.08	1.18 ± 0.13	2.35 ± 0.09	7.51 ± 2.18	0.41 ± 0.05	0.02 ± 0.01	0.03 ± 0.01	12.20	11.74	0.46
25	0.11 ± 0.01	0.74 ± 0.06	1.30 ± 0.14	3.54 ± 0.14	8.07 ± 1.66	0.51 ± 0.08	0.02 ± 0.00	0.05 ± 0.01	14.34	13.76	0.58
26	0.07 ± 0.01	0.98 ± 0.05	1.22 ± 0.04	2.50 ± 0.06	8.99 ± 0.70	0.66 ± 0.04	0.02 ± 0.00	0.02 ± 0.00	14.46	13.75	0.70
27	0.15 ± 0.05	1.01 ± 0.31	2.02 ± 0.14	5.00 ± 0.39	16.30 ± 3.25	0.66 ± 0.09	0.03 ± 0.01	0.03 ± 0.00	25.20	24.48	0.72
28	0.12 ± 0.03	0.81 ± 0.08	1.36 ± 0.02	2.82 ± 0.11	6.44 ± 1.43	0.42 ± 0.02	0.10 ± 0.02	0.02 ± 0.01	12.09	11.55	0.54
29	0.06 ± 0.00	0.80 ± 0.03	0.89 ± 0.07	1.72 ± 0.03	5.33 ± 1.13	0.42 ± 0.07	0.06 ± 0.03	0.04 ± 0.01	9.32	8.80	0.52
30	0.12 ± 0.01	0.93 ± 0.05	0.97 ± 0.02	3.89 ± 0.18	5.64 ± 1.80	0.41 ± 0.05	0.02 ± 0.00	0.04 ± 0.00	12.04	11.56	0.48
31	0.28 ± 0.03	2.15 ± 0.24	3.21 ± 0.32	4.43 ± 0.20	12.83 ± 0.56	2.17 ± 0.00	0.05 ± 0.01	0.09 ± 0.03	25.23	22.91	2.32
33	0.30 ± 0.05	2.07 ± 0.36	3.01 ± 0.12	6.14 ± 0.45	13.80 ± 2.69	1.06 ± 0.05	0.07 ± 0.02	0.05 ± 0.01	26.51	25.33	1.17

Table S1. *Cont.*

Individual	Ap	Ap-4	Ap-7	K-3	K-3.7	Dt-1	Dt-2	Dt-3	Total Compounds	Total Flavonoids	Total Diterpenes
Group A											
34	0.18 ± 0.04	1.92 ± 0.15	3.17 ± 0.45	6.10 ± 0.55	25.82 ± 4.48	1.07 ± 0.08	0.05 ± 0.01	0.07 ± 0.02	38.37	37.18	1.19
35	0.13 ± 0.02	1.39 ± 0.12	2.04 ± 0.11	4.58 ± 0.18	14.30 ± 1.57	0.82 ± 0.02	0.04 ± 0.00	0.03 ± 0.01	23.33	22.44	0.89
36	0.15 ± 0.04	2.32 ± 0.28	2.81 ± 0.35	3.84 ± 0.19	11.15 ± 1.20	1.76 ± 0.04	0.06 ± 0.00	0.04 ± 0.02	22.13	20.27	1.86
38	0.16 ± 0.01	1.93 ± 0.22	2.52 ± 0.23	3.31 ± 0.16	22.65 ± 1.18	0.54 ± 0.03	0.05 ± 0.00	0.11 ± 0.01	31.27	30.57	0.69
39	0.27 ± 0.01	2.93 ± 0.26	2.98 ± 0.18	3.85 ± 0.20	12.72 ± 0.87	1.69 ± 0.02	0.07 ± 0.01	0.08 ± 0.01	24.59	22.75	1.84
40	0.28 ± 0.06	1.73 ± 0.14	2.61 ± 0.19	7.35 ± 0.33	16.91 ± 3.47	1.92 ± 0.08	0.03 ± 0.01	0.09 ± 0.03	30.92	28.89	2.03
47	0.18 ± 0.02	2.02 ± 0.19	2.98 ± 0.32	3.44 ± 0.22	21.01 ± 0.84	1.47 ± 0.03	0.09 ± 0.01	0.11 ± 0.01	31.30	29.63	1.67
48	0.21 ± 0.01	2.10 ± 0.18	3.72 ± 0.30	5.65 ± 0.51	27.08 ± 3.40	1.24 ± 0.10	0.06 ± 0.00	0.10 ± 0.01	40.16	38.76	1.40
49	0.17 ± 0.01	1.98 ± 0.04	2.61 ± 0.14	4.77 ± 0.26	16.88 ± 3.62	1.18 ± 0.03	0.06 ± 0.01	0.03 ± 0.01	27.67	26.41	1.26
51	0.23 ± 0.07	1.72 ± 0.12	3.46 ± 0.15	3.88 ± 0.18	22.16 ± 2.52	1.07 ± 0.03	0.05 ± 0.02	0.08 ± 0.04	32.66	31.45	1.20
56	0.16 ± 0.01	2.83 ± 0.14	3.24 ± 0.16	2.78 ± 0.16	13.95 ± 0.69	1.79 ± 0.05	0.10 ± 0.00	0.05 ± 0.01	24.89	22.95	1.93
57	0.21 ± 0.04	3.23 ± 0.34	3.21 ± 0.12	2.01 ± 0.13	12.83 ± 1.41	1.69 ± 0.03	0.09 ± 0.01	0.09 ± 0.01	23.36	21.49	1.87
59	0.13 ± 0.04	1.86 ± 0.13	2.62 ± 0.17	1.16 ± 0.13	8.81 ± 0.29	1.28 ± 0.03	0.05 ± 0.01	0.02 ± 0.00	15.95	14.59	1.36
60	0.19 ± 0.03	2.01 ± 0.07	2.57 ± 0.19	2.67 ± 0.18	13.43 ± 1.69	0.98 ± 0.11	0.04 ± 0.01	0.02 ± 0.00	21.91	20.87	1.04
61	0.21 ± 0.02	2.39 ± 0.09	2.80 ± 0.08	2.99 ± 0.17	12.02 ± 2.37	1.40 ± 0.05	0.07 ± 0.00	0.03 ± 0.00	21.92	20.42	1.50
62	0.26 ± 0.08	2.69 ± 0.50	3.52 ± 0.18	3.87 ± 0.14	11.68 ± 1.71	1.79 ± 0.04	0.07 ± 0.02	0.06 ± 0.02	23.93	22.01	1.92
65	0.20 ± 0.02	2.76 ± 0.40	3.37 ± 0.15	1.69 ± 0.04	9.89 ± 1.39	1.78 ± 0.04	0.08 ± 0.01	0.10 ± 0.00	19.87	17.91	1.96
66	0.22 ± 0.02	3.32 ± 0.40	3.71 ± 0.38	2.13 ± 0.11	10.63 ± 2.25	1.18 ± 0.01	0.08 ± 0.01	0.10 ± 0.01	21.37	20.01	1.36
67	0.27 ± 0.06	3.38 ± 0.30	4.16 ± 0.57	2.75 ± 0.09	11.09 ± 1.30	1.34 ± 0.02	0.05 ± 0.01	0.06 ± 0.01	23.11	21.66	1.45
68	0.30 ± 0.06	3.61 ± 0.16	4.10 ± 0.22	3.90 ± 0.14	14.00 ± 0.87	1.16 ± 0.03	0.05 ± 0.01	0.08 ± 0.02	27.21	25.92	1.29
70	0.18 ± 0.03	2.01 ± 0.05	2.67 ± 0.24	3.59 ± 0.15	12.43 ± 1.30	1.23 ± 0.01	0.07 ± 0.03	0.04 ± 0.01	22.22	20.88	1.33
71	0.18 ± 0.03	1.93 ± 0.19	2.86 ± 0.14	3.08 ± 0.29	14.95 ± 2.62	1.17 ± 0.07	0.04 ± 0.00	0.10 ± 0.05	24.30	22.99	1.31
72	0.21 ± 0.04	2.67 ± 0.15	3.19 ± 0.20	4.80 ± 0.67	17.55 ± 1.49	1.89 ± 0.02	0.10 ± 0.03	0.11 ± 0.04	30.52	28.42	2.10
74	0.23 ± 0.06	2.40 ± 0.40	2.27 ± 0.23	5.98 ± 0.19	12.48 ± 0.98	0.98 ± 0.02	0.05 ± 0.01	0.06 ± 0.02	24.44	23.35	1.09
78	0.25 ± 0.01	2.10 ± 0.20	2.72 ± 0.17	5.75 ± 0.50	15.45 ± 1.58	1.08 ± 0.17	0.09 ± 0.04	0.08 ± 0.02	27.50	26.26	1.24
81	0.20 ± 0.04	1.60 ± 0.61	2.66 ± 0.09	6.34 ± 0.73	21.18 ± 2.00	1.42 ± 0.05	0.08 ± 0.02	0.07 ± 0.01	33.55	31.99	1.56
82	0.16 ± 0.02	1.91 ± 0.15	2.67 ± 0.20	3.32 ± 0.23	12.82 ± 0.45	1.59 ± 0.09	0.08 ± 0.00	0.04 ± 0.00	22.58	20.88	1.70
85	0.27 ± 0.02	2.35 ± 0.07	2.85 ± 0.10	4.39 ± 0.52	19.35 ± 2.02	1.61 ± 0.01	0.06 ± 0.00	0.05 ± 0.00	30.91	29.19	1.72
88	0.31 ± 0.02	2.37 ± 0.16	3.08 ± 0.07	7.68 ± 0.57	20.15 ± 2.46	1.69 ± 0.02	0.06 ± 0.01	0.03 ± 0.00	35.37	33.60	1.78
89	0.18 ± 0.01	1.90 ± 0.16	2.47 ± 0.20	7.09 ± 0.31	12.48 ± 1.05	0.90 ± 0.02	0.05 ± 0.01	0.11 ± 0.02	25.17	24.12	1.06
93	0.17 ± 0.04	1.75 ± 0.10	2.56 ± 0.13	4.41 ± 0.12	20.52 ± 3.27	0.66 ± 0.05	0.04 ± 0.00	0.09 ± 0.02	30.19	29.40	0.78
94	0.19 ± 0.04	1.96 ± 0.10	3.70 ± 0.25	4.32 ± 0.48	29.97 ± 3.13	1.06 ± 0.03	0.04 ± 0.01	0.07 ± 0.02	41.31	40.14	1.17
96	0.19 ± 0.03	1.97 ± 0.12	2.88 ± 0.14	2.14 ± 0.16	8.30 ± 0.47	0.95 ± 0.06	0.05 ± 0.01	0.11 ± 0.01	16.60	15.48	1.11
97	0.13 ± 0.00	1.64 ± 0.20	2.70 ± 0.15	2.42 ± 0.15	22.01 ± 1.50	0.85 ± 0.02	0.04 ± 0.01	0.06 ± 0.02	29.85	28.91	0.94
99	0.24 ± 0.06	1.88 ± 0.16	2.61 ± 1.16	2.52 ± 0.18	14.74 ± 0.09	2.03 ± 0.08	0.07 ± 0.03	0.07 ± 0.01	24.15	21.98	2.17
100	0.24 ± 0.04	2.17 ± 0.24	2.94 ± 0.07	5.52 ± 0.36	19.01 ± 0.91	0.89 ± 0.01	0.03 ± 0.00	0.08 ± 0.02	30.89	29.89	1.01

Table S1. *Cont.*

Individual	Ap	Ap-4	Ap-7	K-3	K-3.7	Dt-1	Dt-2	Dt-3	Total Compounds	Total Flavonoids	Total Diterpenes
Group B											
41	0.38 ± 0.07	2.76 ± 0.22	3.57 ± 0.23	8.66 ± 0.66	26.85 ± 1.89	1.16 ± 0.03	0.07 ± 0.01	0.08 ± 0.03	43.52	42.21	1.31
42	0.42 ± 0.05	2.52 ± 0.09	4.58 ± 0.02	11.34 ± 0.72	39.49 ± 0.93	1.62 ± 0.03	0.06 ± 0.02	0.14 ± 0.01	60.17	58.35	1.82
52	0.49 ± 0.03	2.71 ± 0.32	5.44 ± 0.19	8.93 ± 1.47	29.88 ± 1.87	1.42 ± 0.02	0.12 ± 0.02	0.03 ± 0.00	49.03	47.45	1.58
73	0.31 ± 0.10	2.13 ± 0.16	3.79 ± 0.11	11.04 ± 3.39	37.40 ± 4.89	0.96 ± 0.02	0.11 ± 0.01	0.04 ± 0.00	55.78	54.67	1.11
75	0.50 ± 0.06	3.26 ± 0.20	5.41 ± 0.70	14.57 ± 2.27	30.56 ± 5.39	1.46 ± 0.02	0.07 ± 0.01	0.08 ± 0.01	55.91	54.30	1.62
79	0.61 ± 0.09	3.75 ± 0.31	5.01 ± 0.54	12.44 ± 2.41	45.64 ± 3.23	1.61 ± 0.09	0.10 ± 0.02	0.09 ± 0.02	69.25	67.45	1.80
83	0.49 ± 0.07	2.87 ± 0.14	5.48 ± 0.53	10.13 ± 4.73	30.32 ± 5.43	2.06 ± 0.05	0.07 ± 0.01	0.08 ± 0.04	51.51	49.29	2.22
87	0.39 ± 0.09	2.45 ± 0.31	4.86 ± 0.38	9.77 ± 2.86	30.73 ± 5.09	1.17 ± 0.02	0.05 ± 0.01	0.11 ± 0.02	49.52	48.19	1.34
Group C											
1	0.15 ± 0.05	0.96 ± 0.08	2.17 ± 0.10	3.09 ± 1.94	25.92 ± 3.73	1.23 ± 0.08	0.07 ± 0.04	0.18 ± 0.07	33.78	32.29	1.48
2	0.19 ± 0.04	0.91 ± 0.08	2.69 ± 0.04	3.65 ± 0.35	23.72 ± 4.18	1.22 ± 0.01	0.07 ± 0.01	0.16 ± 0.06	32.62	31.17	1.45
84	0.30 ± 0.08	2.06 ± 0.20	3.45 ± 0.19	5.87 ± 0.36	25.87 ± 3.77	1.80 ± 0.02	0.09 ± 0.01	0.19 ± 0.02	39.63	37.55	2.08
86	0.27 ± 0.01	3.00 ± 0.08	3.75 ± 0.21	4.08 ± 0.29	25.76 ± 1.85	2.33 ± 0.02	0.08 ± 0.00	0.18 ± 0.02	39.44	36.85	2.58
90	0.29 ± 0.03	2.46 ± 0.16	3.24 ± 0.14	7.18 ± 1.21	38.43 ± 2.48	1.82 ± 0.03	0.09 ± 0.01	0.19 ± 0.03	53.70	51.59	2.11
91	0.31 ± 0.02	1.89 ± 0.08	5.05 ± 0.31	2.46 ± 0.16	24.52 ± 2.34	1.19 ± 0.02	0.05 ± 0.01	0.16 ± 0.03	35.64	34.24	1.40
92	0.24 ± 0.06	2.89 ± 0.06	3.28 ± 0.28	3.40 ± 1.44	22.70 ± 2.91	0.77 ± 0.02	0.04 ± 0.01	0.23 ± 0.06	33.55	32.51	1.03
98	0.22 ± 0.02	1.75 ± 0.07	3.27 ± 0.26	4.54 ± 0.56	28.70 ± 4.20	0.85 ± 0.04	0.05 ± 0.00	0.18 ± 0.03	39.56	38.48	1.08
Group D											
32	0.31 ± 0.04	1.98 ± 0.06	4.63 ± 0.12	7.42 ± 1.36	28.47 ± 0.61	1.80 ± 0.03	0.07 ± 0.01	0.14 ± 0.04	44.82	42.81	2.01
37	0.20 ± 0.06	2.38 ± 0.15	3.00 ± 0.19	4.60 ± 0.12	13.72 ± 2.02	2.94 ± 0.08	0.06 ± 0.01	0.12 ± 0.03	27.03	23.91	3.12
43	0.32 ± 0.04	2.10 ± 0.09	4.62 ± 0.25	7.56 ± 3.83	31.27 ± 1.31	2.11 ± 0.02	0.08 ± 0.01	0.09 ± 0.04	48.15	45.87	2.28
44	0.34 ± 0.02	2.42 ± 0.10	4.52 ± 0.27	4.75 ± 1.10	23.82 ± 3.90	1.93 ± 0.03	0.06 ± 0.04	0.11 ± 0.04	37.94	35.85	2.09
45	0.28 ± 0.02	2.81 ± 0.11	4.07 ± 0.02	4.34 ± 0.66	24.39 ± 1.57	2.03 ± 0.11	0.10 ± 0.01	0.09 ± 0.01	38.11	35.89	2.22
46	0.38 ± 0.01	3.09 ± 0.06	4.54 ± 0.07	4.76 ± 0.34	23.59 ± 1.70	2.23 ± 0.09	0.11 ± 0.01	0.13 ± 0.02	38.81	36.35	2.46
50	0.29 ± 0.01	2.91 ± 0.01	3.93 ± 0.20	2.84 ± 0.37	20.66 ± 4.32	2.36 ± 0.12	0.11 ± 0.01	0.07 ± 0.01	33.17	30.64	2.53
53	0.30 ± 0.05	2.97 ± 0.33	3.60 ± 0.23	7.47 ± 1.36	26.85 ± 4.56	2.65 ± 0.07	0.13 ± 0.04	0.07 ± 0.02	44.04	41.19	2.85
54	0.28 ± 0.00	3.39 ± 0.38	3.77 ± 0.31	3.79 ± 0.84	13.04 ± 1.23	2.07 ± 0.03	0.12 ± 0.04	0.06 ± 0.01	26.52	24.28	2.25
55	0.40 ± 0.02	3.65 ± 0.28	4.39 ± 0.13	11.07 ± 0.69	19.74 ± 1.07	2.00 ± 0.02	0.08 ± 0.01	0.15 ± 0.01	41.47	39.25	2.23
58	0.27 ± 0.03	2.98 ± 0.14	3.37 ± 0.10	3.29 ± 0.70	17.70 ± 3.15	2.50 ± 0.01	0.10 ± 0.01	0.05 ± 0.00	30.27	27.61	2.65
63	0.28 ± 0.03	3.63 ± 0.20	4.01 ± 0.24	4.18 ± 0.85	13.37 ± 2.21	1.82 ± 0.05	0.08 ± 0.01	0.13 ± 0.01	27.51	25.48	2.03
64	0.34 ± 0.07	3.46 ± 0.24	4.31 ± 0.57	4.92 ± 0.91	18.83 ± 3.74	1.20 ± 0.03	0.10 ± 0.02	0.09 ± 0.02	33.25	31.86	1.38
69	0.36 ± 0.09	3.23 ± 0.15	3.92 ± 0.19	6.17 ± 1.92	18.35 ± 4.28	1.42 ± 0.06	0.07 ± 0.01	0.13 ± 0.03	33.65	32.03	1.62
76	0.49 ± 0.10	2.98 ± 0.11	4.42 ± 0.50	8.21 ± 3.32	20.13 ± 3.29	1.51 ± 0.03	0.08 ± 0.03	0.11 ± 0.02	37.92	36.22	1.69
77	0.36 ± 0.06	2.98 ± 0.37	3.80 ± 0.28	6.04 ± 2.96	21.00 ± 2.37	1.35 ± 0.03	0.08 ± 0.01	0.08 ± 0.03	35.68	34.17	1.50
80	0.30 ± 0.08	2.73 ± 0.14	3.79 ± 0.11	7.66 ± 2.26	20.27 ± 3.43	1.49 ± 0.06	0.08 ± 0.02	0.11 ± 0.04	36.44	34.75	1.68
95	0.36 ± 0.02	3.35 ± 0.36	4.40 ± 0.28	7.55 ± 0.44	35.26 ± 1.58	2.07 ± 0.02	0.12 ± 0.01	0.09 ± 0.01	53.19	50.92	2.27
